# Association of early tracheostomy with the mortality and complications of adult patients in intensive care unit

**DOI:** 10.1186/2197-425X-3-S1-A673

**Published:** 2015-10-01

**Authors:** MF Aguilar Arzapalo, VL Avendaño, AM Escalante, J Gongora Mukul, MA Cetina Camara, L Soberanes Ramirez, YucaCrit

**Affiliations:** Universidad Autónoma de Yucatan, Critical Care Department, Mérida, Mexico; Universidad Autónoma de Yucatan, Mérida, Mexico

## Introduction

Prolonged intubation has been associated with increased mortality and morbidity of patients in critical condition. There is debate over whether early tracheostomy within 7 days, should be or not performed during the stay in the intensive care unit and its relationship with improvement in mortality and morbidity at discharge and 28 days. Recently, a large controlled clinical trial to determine that there is no difference between early or late tracheostomy. However a subsequent meta-analysis determined that if there is improvement in the realization of early tracheostomy.

## Objectives

Determine if there is association between the realization of early tracheostomy in the mortality and complications of adult patients in the intensive care unit at discharge and 28 days.

## Methods

Historical cohort study of risk and association. Data were retrospectively collected for consecutive adult patients admitted to Agustin O´Horan Hospital ICU Merida Mexico, between January 2011 and July 2014, who underwent inpatient medical treatment using electronic files.

## Results

The dataset consisted of 936 medical files, 853 being eligible. Early tracheostomy was quite common, with an incidence of 49.2%. Patients were propensity matched based on their association with death and early tracheostomy. Of the 853 patients collected, patients with early tracheostomy after admission (n = 419, 49.2%), and late tracheostomy (n=434, 50.8%) were matched in two groups. These two groups were well balanced with respect to all variables collected. The early tracheostomy group had at decreased risk of mortality at ICU discharge, relative risk ratio: 0.78; 95% CI (0.78-.97) with a decreased risk of 22% vs the late tracheostomy group who had a increase risk of death at ICU discharge, relative risk ratio: 1.15; 95% CI (1.05-1.17). No significant differences in mortality were found at 28 days and no diferences in complications. Early tracheostomy was associated with decreased mortality whitout additional complications; this association was statistically important.

## Conclusions

This retrospective cohort trial demonstrates an association between early tracheostomy and better ICU outcome (mortality). Additional studies are required to demonstrate a causal relationship between these variables.

## Grant Acknowledgment

To medical research department of OHoran Hospital

**Figure 1:**
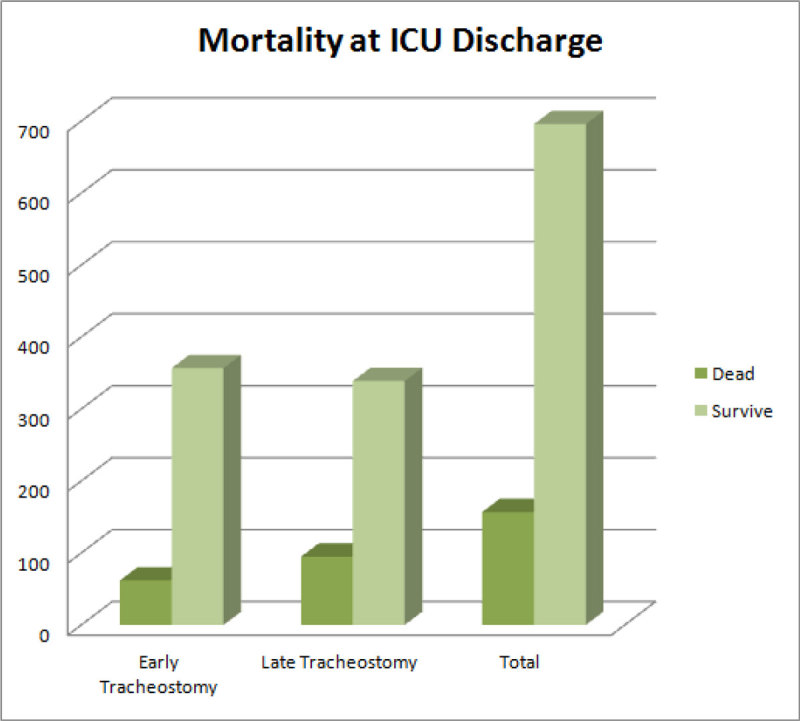
**Mortality at ICU Discharge.**

**Figure 2:**
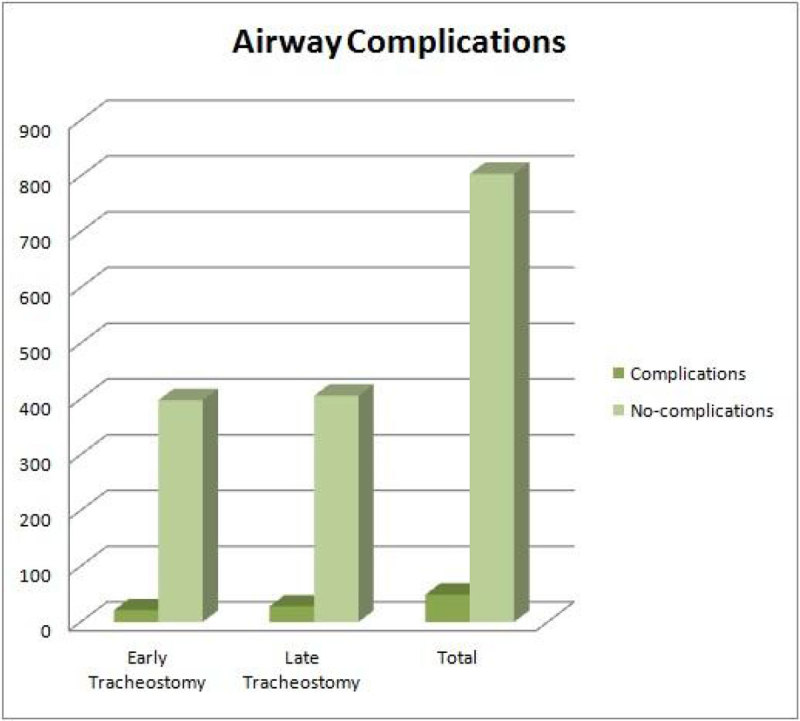
**Airway Complications.**
